# Do overweight children necessarily make overweight adults? Repeated cross sectional annual nationwide survey of Japanese girls and women over nearly six decades

**DOI:** 10.1136/bmj.a802

**Published:** 2008-08-21

**Authors:** Ikuko Funatogawa, Takashi Funatogawa, Eiji Yano

**Affiliations:** 1 Department of Hygiene and Public Health, Teikyo University School of Medicine, Tokyo 173-8605, Japan; 2 Clinical Research Planning Department, Chugai Pharmaceutical Company, Tokyo 103-8324

## Abstract

**Objective** To compare growth curves of body mass index from children to adolescents, and then to young adults, in Japanese girls and women in birth cohorts born from 1930 to 1999.

**Design** Retrospective repeated cross sectional annual nationwide surveys (national nutrition survey, Japan) carried out from 1948 to 2005.

**Setting** Japan.

**Participants** 76 635 females from 1 to 25 years of age.

**Main outcome measure** Body mass index.

**Results** Generally, body mass index decreased in preschool children (2-5 years), increased in children (6-12 years) and adolescents (13-18 years), and slightly decreased in young adults (19-25 years) in these Japanese females. However, the curves differed among birth cohorts. More recent cohorts were more overweight as children but thinner as young women. The increments in body mass index in early childhood were larger in more recent cohorts than in older cohorts. However, the increments in body mass index in adolescents were smaller and the decrease in body mass index in young adults started earlier, with lower peak values in more recent cohorts than in older cohorts. The decrements in body mass index in young adults were similar in all birth cohorts.

**Conclusions** An overweight birth cohort in childhood does not necessarily continue to be overweight in young adulthood. Not only secular trends in body mass index at fixed ages but also growth curves for wide age ranges by birth cohorts should be considered to study obesity and thinness. Growth curves by birth cohorts were produced by a repeated cross sectional annual survey over nearly six decades.

## Introduction

The prevalence of childhood obesity is increasing rapidly worldwide.^1^ ^2^ Recently, obesity in childhood has become an important public health concern because early obesity is considered to result in obesity in later life and a higher prevalence of obesity related disorders.^1^ Thus, monitoring changes in body mass index over time is helpful.^3^ Several papers have reported on secular trends in childhood body mass index, compared body mass index values at fixed ages, and described secular trends in the prevalence of obesity and overweight, defined by body mass index.^2^ ^4^ ^5^ Most of these studies have shown increasing body mass index and prevalence of obesity in children.

In Japan, as in most nations, the mean body mass index of girls aged 6-14 years increased between 1976 and 2000, and prevalence increased from 1.2% to 2.9% for obesity and from 10.1% to 17.2% for overweight and obesity (International Obesity Task Force definition^6^).^2^ ^7^ However, the mean body mass index of young women aged 15-29 has decreased.^8^ ^9^ These results indicate the possibility that a birth cohort that might have been more overweight in childhood became thinner as young adults. That is, at a population level, a more overweight birth cohort in childhood may not necessarily continue to be more overweight as young adults. However, the trajectories of body mass index from children to young adults by birth cohorts at a national level have not been studied, and no reported study has compared growth curves among multiple birth cohorts in a nation.

Values of body mass index from childhood to adulthood have been reported.^10^ ^11^ However, most of these data were based on cross sectional studies. When estimating the growth in a birth cohort, the growth curve may be distorted by cohort effects.^12^ ^13^ ^14^ The shapes of growth curves from childhood to adulthood by birth cohorts have not been examined at a national level.

Frequent observations taken over more than two decades are necessary to make a growth curve from childhood to young adulthood of a cohort. Furthermore, additional decades are needed to compare growth curves in body mass index among multiple birth cohorts born in different decades. In this paper, we provide the growth curves of body mass index by birth cohorts in Japanese girls and women aged 1-25 years to quantitatively assess differences in growth curves. We focused on females, because the trend of young Japanese women being thinner is striking compared with the opposite trend of most other nations as well as young Japanese men.^8^ We used nationwide data from the national nutrition survey, Japan, gathered annually between 1948 and 2005.

## Methods

The national nutrition survey, Japan, has been done annually since 1948 with large random samples of the Japanese population. It is the only data source for annual anthropometric measurements of Japanese adults, although other data sources exist for children. Details of the survey have been described elsewhere.^15^ ^16^ The survey covers approximately 5000 households in 300 randomly selected census units, as defined by the Ministry of Health, Labour and Welfare, Japan. The data are regarded as representative of the Japanese population. Participants were gathered locally, and height and weight were measured.

We used data from the 1948-2005 surveys for females aged 1-25 years who were born from 1930 to 1999. Because the survey was not done in 1974, 57 surveys were carried out during the past 58 years. We used mean values of height and weight by age because no individual data exist for the early survey years (1948-72). We defined the participants aged 25 years old in 2005 as the 1980 birth cohort. We grouped the birth cohorts by decade, giving seven cohort groups from the 1930s to the 1990s. The table[Table tbl1] shows a summary of birth cohort groups. Only the third, fourth, and fifth groups (1950s, 1960s, and 1970s) have all the data from age 1 to 25 years. The only data used for the 1930s cohort groups were from 18 year olds, and only data from 8 year olds were used for the 1940s cohort group. Likewise, data used for the 1980s cohort group were up to age 16, and those used for the 1990s cohort group were up to age 6. The total sample size of the 1972-2005 surveys was 76 635. However, sample size information for the 1948-71 surveys is missing. The average number of participants in each age by cohort group was 986.

**Table tbl1:** Birth cohort groups of females in national nutrition survey, Japan, 1948-2005 (n=76 635)

Cohort groups	Birth years	Age range (years)	Years of measurement			Sample size	
			First cohort	Last cohort		1-12 years old	13-25 years old
1930s	1930-9	18-25	1948-55	1957-64		Not analysed	Does not exist
1940s	1940-9	8-25	1948-65	1957-73		Does not exist	717
1950s	1950-9	1-25	1951-75	1960-84		Does not exist	7 832
1960s	1960-9	1-25	1961-85	1970-94		8 710	12 271
1970s	1970-9	1-25	1971-95	1980-2004		18 538	10 805
1980s	1980-9	1-16	1981-96	1990-2005		11 627	2 558
1990s	1990-9	1-6	1991-6	2000-5		3 577	Not analysed

Sample size information missing for surveys done in 1948-71. Survey not done in 1974.

### Statistical analysis

We calculated the body mass index for each survey year as the mean weight (kg) divided by the square of the mean height (m). We estimated the mean values of body mass index by age and birth cohort group. We calculated the annual change in body mass index by subtracting the previous year’s body mass index from the current one, by birth cohorts. We fitted non-linear curves by cohort group to the annual change data by using cubic smoothing spline curves. We set the degrees of freedom per age to be identical among cohort groups; they were the average of the values selected by the generalised cross validation function. We used the generalised additive model procedure of the SAS 9.1 statistical package.

## Results

Figure 1 shows the growth curves of the mean body mass index in the seven birth cohort groups of Japanese girls and women aged 1-25 years born from the 1930s to the 1990s. The patterns of growth curves are similar; they decrease in preschool children (2-5 years), then increase in children (6-12 years) and adolescents (13-18 years), and slightly decrease in young adults (19-25 years). However, the exact values differed among the birth cohorts. The mean value of body mass index was higher in more recent cohort groups during childhood (6-12 years) and early adolescence (13-14 years) than in older cohort groups. However, the growth curves intersect at middle adolescence (15-16 years), and the body mass index value was lower in more recent cohort groups during late adolescence (17-18 years) and young adults (19-25 years).

**Figure fig1:**
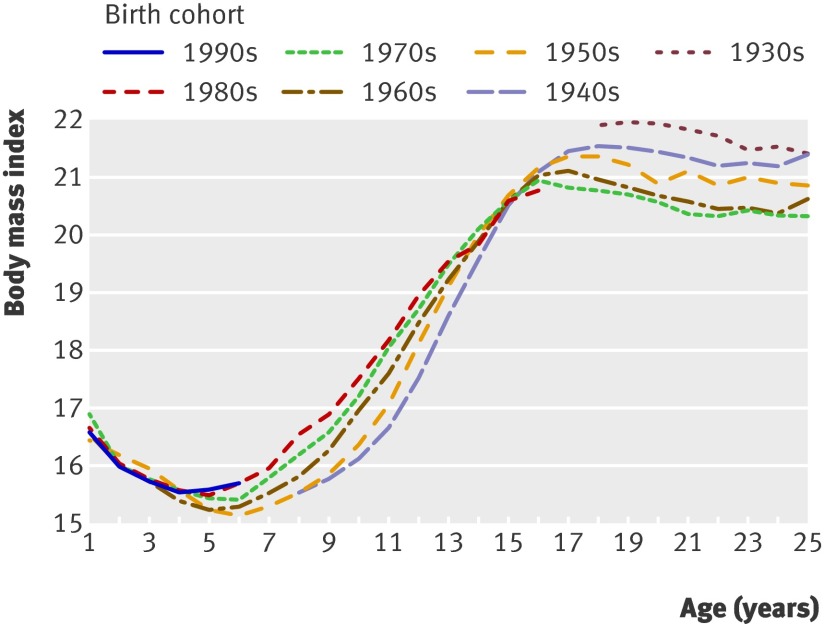
**Fig 1** Growth curves of body mass index by female birth cohorts (1930s to 1990s), from national nutrition survey, Japan, 1948-2005

Figure 2 shows the annual change curves for body mass index. The age at which the curve crosses zero in childhood corresponds to the age at adiposity rebound, and this age was earlier in more recent cohort groups. The increment in body mass index was larger in more recent cohort groups during early childhood (6-9 years) than in older cohorts. However, the annual change curves intersect at late childhood (10-12 years), and the increments in body mass index in adolescents (13-18 years) were smaller in more recent cohort groups than in older ones. The slight decrease in body mass index in young adults (19-25 years) started earlier in more recent cohort groups, but the decrements in body mass index in young adults were similar across all birth cohorts.

**Figure fig2:**
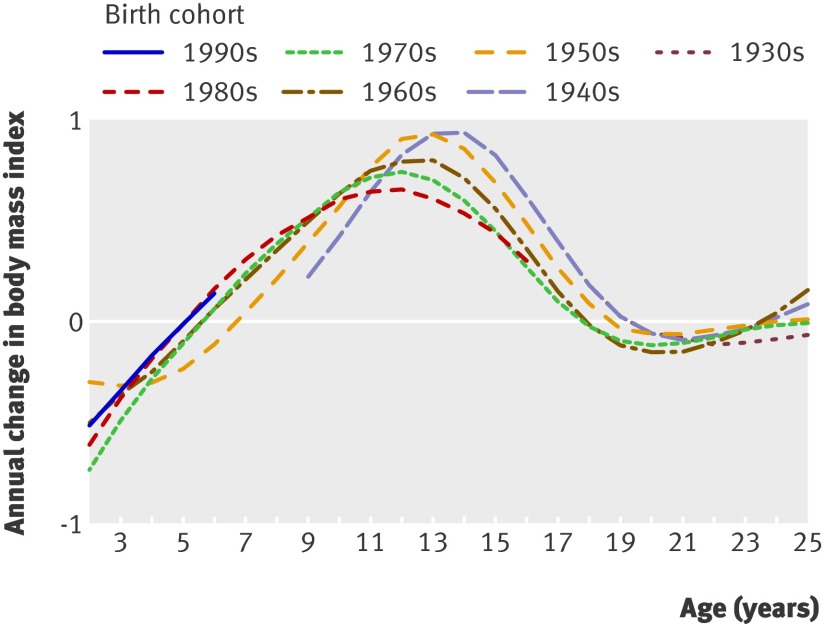
**Fig 2** Annual change in body mass index by female birth cohorts (1930s to 1990s), from national nutrition survey, Japan, 1948-2005

## Discussion

More recent Japanese female cohorts were relatively more overweight in childhood than older cohorts, but they grew to be relatively thin as young adults. Recently, obesity in childhood has become an important public health concern worldwide because early obesity is considered to result in obesity in later life, as well as a high prevalence of obesity related disorders.^1^ However, our result provides a counter-example at the population level; that is, an overweight birth cohort in childhood does not necessarily continue to be overweight in adulthood. This should not be taken as rejecting the possible relation between early obesity and obesity in later life at the individual level. To our knowledge, this is the first report to show the growth curves of body mass index from childhood to young adulthood by birth cohort. Our results show the importance of observing growth curves of body mass index by birth cohort and monitoring a wide span of ages, not only in children but also in adults.

### Body mass index in later life

Although our study is restricted to the period from childhood to young adulthood, the body mass index value in later life for each birth cohort is also important. Body mass index is generally considered to increase with age. In fact, the body mass index value of the 1936-45 birth cohorts at 20 years old was slightly less than 22, and it became 23.6 at 60-69 years in the 2005 survey. The prevalences of overweight (body mass index ≥25) and thinness (body mass index <18.5) in 2005 were 29.0% and 5.4%.^16^ As the members of these cohorts have become older, they too have a problem with obesity despite their ideal body mass index value as young adults. The body mass index of the 1976-85 birth cohorts at age 20-29 in the 2005 survey was 20.5, and the prevalences of overweight and thinness were 5.6% and 22.6%.^16^ The prevalence of overweight (International Obesity Task Force definition^6^) in these cohorts at age 9-11 was 12.8% in 1986-90 and 14.8% in 1991-5; it was greater than the prevalence at age 9-11 in 1976-80 (9.3%).^7^ However, whether these cohorts will be continuously thin as they age in future is not known. Furthermore, our study is restricted to 6 years of age for the 1990s cohort groups and 16 years for the 1980s cohort groups. The 1990s cohort group has not yet grown up, and whether they will grow up thinner is not known.

### Increasing body mass index in childhood and thinness in young women

The national nutrition survey, Japan, was started after the second world war to assess nutritional conditions. Japan experienced severe food shortages until 1948.^16^ The 1930s and 1940s cohort groups had experienced the war, food shortages, or both, and these would cause the low body mass index seen in childhood and early adolescence. After the war, as Japan was rapidly developed in a westernised and industrialised manner, intake of fat and animal protein quickly increased until about 1975.^16^ ^17^ In addition, sedentary lifestyle became more prevalent.^17^ These are possible causes of the increased body mass index seen in childhood.

In Japan, the prevalence of being underweight has increased among young women aged 15-29, and excessive thinness (body mass index <17), which had a prevalence of 4.2% in 1996-2000, has become a public concern.^9^ On the other hand, the prevalence of being underweight decreased among Japanese girls aged 10-14.^18^ These changes, in opposite directions, in the two age groups were consistent with our results (fig 1). Our study also showed that the annual changes in body mass index by birth cohort, and the increment in body mass index during adolescence (13-18 years), had decreased in more recent cohorts compared with older cohorts (fig 2). Thus, the problem of excessive thinness in young women may have already started in early adolescence (13-14 years). In the national nutrition survey, Japan, in 2002, more than half of women aged 15-29 regarded themselves as being overweight or slightly overweight.^19^ This percentage was higher than that in 1979, although the body mass index was lower. The major reason behind this self perception as being overweight was “comparison with other people.”^19^ The survey data showed that 64% of women aged 15-19 and 54% of those aged 20-29 were attempting to lose weight.^19^ The practice of excessive dieting to become slim is seen among teenage girls in Japan.^20^ The effects of behaviour to lose weight may appear earlier in annual changes and then be seen in the mean body mass index later. Because body mass index changes dynamically in childhood, secular trends should be interpreted carefully. Annual changes provide complementary information.

### Repeated cross sectional survey

A specific feature of this study was the use of a repeated cross sectional survey with random sampling to show the growth curve by birth cohort. In a cross sectional study each child is observed once, and in a longitudinal study each child is observed several times. In a repeated cross sectional survey each child is observed once, but each birth cohort is studied several times. Thus, we can infer the growth curve of a birth cohort from a repeated cross sectional survey. A repeated cross sectional survey is usually reported as a cross sectional growth curve by survey year,^21^ but such a cross sectional growth curve is different from the growth curve of a birth cohort when a cohort effect occurs. In a cross sectional growth curve, we see combined effects of age and cohort. When interpreting body mass index values over a long time, such as more than a decade, as we did here, the cohort effect should be considered. A repeated annual survey is suited to monitoring the growth curve by birth cohort, because growth curves can be produced and checked for gradual changes by generation.

The weakness of a repeated cross sectional survey, compared with a longitudinal study, is that all inferences are described in terms of population averages, and the variability of growth curves among individuals and the effects of covariates cannot be inferred. Furthermore, when the curve is non-linear, individual growth curves can differ greatly from that of the population average. The shapes of the growth curve for height and weight obtained by following a single person longitudinally and that obtained by population average are well known to be different because the timing of the growth spurt, the sharp increase in growth, varies greatly between individual people.^22^ A repeated cross sectional survey and a longitudinal study should be considered as being complementary.

### Cut offs for prevalence

Recently, the International Obesity Task Force proposed sex and age specific international body mass index cut offs for overweight, obesity, and thinness in childhood and adolescence, from 2 to 18 years.^6^ ^23^ These cut offs were developed to help provide internationally comparable prevalences in children and are based on nationally representative datasets from six countries gathered from 1963 to 1993. This means that the current definitions of overweight, obesity, and thinness are based on the distribution of body mass index in past birth cohorts at fixed ages. If the pattern of growth curves has changed with generations, it should affect the prevalence of obesity and thinness. If the timing of growth is accelerated, the prevalence of obesity should increase in childhood, as our results suggest in Japanese girls. Indeed, a study that used another nationwide survey of Japanese children aged 5-17 showed that the age at which the greatest increase in height occurred became earlier, and the increasing trends of body mass index in childhood were reduced by seeing the body mass index at this age.^24^ However, these data showed that, even at this age, the body mass index has increased by about 1 unit over the past 40 years. We need careful monitoring of the prevalence at later ages for recent cohorts. When long term health promotions are planned or assessed, policy makers need to look at changes in birth cohorts.

### Other nations

Considering the worldwide epidemic of obesity in adults, prevention of obesity in the earlier stages of life is an important public health priority. In Denmark, the increased prevalence of obesity was seen in the same male birth cohorts at both childhood (7-11 years) and young adulthood (19 years); this suggests that early life is a susceptible period for the development of obesity.^25^ However, young Japanese women tended to be thinner despite a higher body mass index in childhood. Whether this phenomenon is specific to Japanese women or holds true for other nations is not known. In fact, the intersect of the growth curve at middle adolescence is not seen in Japanese males from the same dataset, although the rate of recent increase in body mass index was smaller in young adults than in children. General patterns of body mass index after about 17 years of age differed between men and women. The body mass index of young women slightly decreased in all cohort groups, whereas that of young men increased. Whether this slight decrease in young women in each cohort is seen in other nations is also unknown. The body mass index in young Korean women is as low as that of Japanese women and also shows the decrease during the ages of 20-25 for cross sectional data; however, this decrease is not seen in each birth cohort.^26^ Currently, growth curves of mean body mass index or prevalence by birth cohorts in each nation for a wide range of ages, childhood to old age, are unclear and need further study.

### Conclusions

We have shown that an overweight birth cohort in childhood does not necessarily continue to be overweight as young adults. Monitoring growth curves by birth cohort is important in studying obesity and thinness for public health. Values of body mass index from childhood to later life by birth cohort should be examined. For this purpose, a repeated cross sectional survey is suitable. Values of body mass index and annual changes in body mass index are important because body mass index changes dynamically during childhood and adolescence.

**What is already known on this topic**

A growing global epidemic of childhood obesity is occurring
The mean body mass index in Japanese females has increased in childhood in recent decades but decreased in young adulthood; the effect of birth cohorts on this phenomenon is unclear

**What this study adds**

Growth curves by birth cohorts were produced by a repeated cross sectional annual survey over nearly six decades
More recent cohorts of Japanese females were more overweight in childhood but thinner in young adulthood
Growth curves for a wide age range by birth cohort should be considered in studying obesity and thinness

## Notes

Ethical approval: Not needed.

Provenance and peer review: Not commissioned; externally peer reviewed.

## Notes

**Cite this as:** *BMJ* 2008;337:a802
